# Direct evidence for a pressure-induced nodal superconducting gap in the Ba_0.65_Rb_0.35_Fe_2_As_2_ superconductor

**DOI:** 10.1038/ncomms9863

**Published:** 2015-11-09

**Authors:** Z. Guguchia, A. Amato, J. Kang, H. Luetkens, P. K. Biswas, G. Prando, F. von Rohr, Z. Bukowski, A. Shengelaya, H. Keller, E. Morenzoni, Rafael M. Fernandes, R. Khasanov

**Affiliations:** 1 Laboratory for Muon Spin Spectroscopy, Paul Scherrer Institute, CH-5232 Villigen, Switzerland; 2 School of Physics and Astronomy, University of Minnesota, Minneapolis, Minnesota 55455, USA; 3 Leibniz-Institut für Festkörper- und Werkstoffforschung (IFW) Dresden, D-01171 Dresden, Germany; 4 Physik-Institut der Universität Zürich, Winterthurerstrasse 190, CH-8057 Zürich, Switzerland; 5 Institute of Low Temperature and Structure Research, Polish Academy of Sciences, 50-422 Wroclaw, Poland; 6 Department of Physics, Tbilisi State University, Chavchavadze 3, GE-0128 Tbilisi, Georgia

## Abstract

The superconducting gap structure in iron-based high-temperature superconductors (Fe-HTSs) is non-universal. In contrast to other unconventional superconductors, in the Fe-HTSs both *d*-wave and extended *s*-wave pairing symmetries are close in energy. Probing the proximity between these very different superconducting states and identifying experimental parameters that can tune them is of central interest. Here we report high-pressure muon spin rotation experiments on the temperature-dependent magnetic penetration depth in the optimally doped nodeless *s*-wave Fe-HTS Ba_0.65_Rb_0.35_Fe_2_As_2_. Upon pressure, a strong decrease of the penetration depth in the zero-temperature limit is observed, while the superconducting transition temperature remains nearly constant. More importantly, the low-temperature behaviour of the inverse-squared magnetic penetration depth, which is a direct measure of the superfluid density, changes qualitatively from an exponential saturation at zero pressure to a linear-in-temperature behaviour at higher pressures, indicating that hydrostatic pressure promotes the appearance of nodes in the superconducting gap.

## Abstract (web summary)

Evidence suggests that the superconducting gap structures of Fe-based high temperature superconductors are highly sensitive to material parameters. Here, the authors use muon spin rotation to evidence the appearance of superconducting nodes in optimally-doped Ba_0.65_Rb_0.35_Fe_2_As_2_ under hydrostatic pressure.

## Graphical abstract

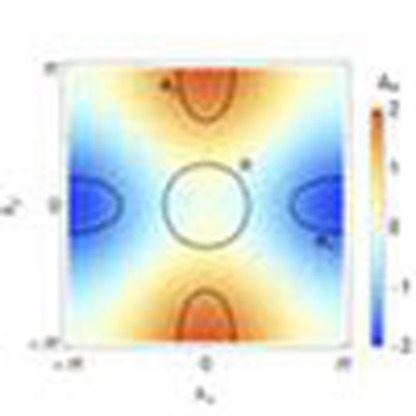

After 6 years of intensive research on the Fe-based high-temperature superconductors (Fe-HTSs), no consensus on a universal gap structure has been reached. There is evidence that small differences in electronic or structural properties can lead to a strong diversity in the superconducting (SC) gap structure. On the one hand, nodeless isotropic gap functions were observed in optimally doped Ba_1−*x*_K_*x*_Fe_2_As_2_, Ba_1−*x*_Rb_*x*_Fe_2_As_2_ and BaFe_2−*x*_Ni_*x*_As_2_ as well as in BaFe_2−*x*_Co_*x*_As_2_, K_*x*_Fe_2−*y*_Se_2_ and FeTe_1−*x*_Se_*x*_ (refs 1, 2, 3, 4, 5, 6, 7, 8). On the other hand, signatures of nodal SC gaps were reported in LaOFeP, LiFeP, KFe_2_As_2_, BaFe_2_(As_1−*x*_P_*x*_)_2_, BaFe_2−*x*_Ru_*x*_As_2_, FeSe as well as in overdoped Ba_1−*x*_K_*x*_Fe_2_As_2_ and BaFe_2−*x*_Ni_*x*_As_2_ (refs 7, 9, 10, 11, 12, 13, 14, 15, 16, 17). Understanding what parameters of the systems control the different SC gap structures observed experimentally is paramount to elucidate the microscopic pairing mechanism in the Fe-HTSs and, more generally, to provide a deeper understanding of the phenomenon of high-temperature superconductivity. On the theoretical front, it has been proposed that both the *s*^+−^-wave and *d*-wave states are close competitors for the SC ground state^18^,^19^,^20^,^21^,^22^,^23^,^24^,^25^. Although the former generally wins, it has been pointed out that a *d*-wave state may be realized on removing electron or hole pockets. On the experimental front, a sub-leading *d*-wave collective mode was observed by Raman experiments inside the fully gapped SC state of optimally doped Ba_1−*x*_K_*x*_Fe_2_As_2_ (refs 26, 27). In KFe_2_As_2_, a change of the SC pairing symmetry by hydrostatic pressure has been recently proposed, based on the V-shaped pressure dependence of *T*_c_ (ref. 28). However, no direct experimental evidence for a pressure-induced change of either the SC gap symmetry or the SC gap structure in the Fe-HTSs has been reported until now.

Measurements of the magnetic penetration depth *λ*, which is one of the fundamental parameters of a superconductor, since it is related to the superfluid density *n*_s_ via 1/*λ*^2^=*μ*_0_*e*^2^*n*_s_/m* (where m* is the effective mass), are a sensitive tool to study multiband superconductivity. Most importantly, the temperature dependence of *λ* is particularly sensitive to the presence of SC nodes: while in a fully gapped SC Δ*λ*^−2^(*T*)≡*λ*^−2^(0)−*λ*^−2^(*T*) vanishes exponentially at low *T*, in a nodal SC it vanishes as a power of *T*. The muon spin rotation (μSR) technique provides a powerful tool to measure *λ* in type II superconductors^29^. A μSR experiment in the vortex state of a type II superconductor allows the determination of *λ* in the bulk of the sample, in contrast to many techniques that probe *λ* only near the surface.

For the compound Ba_0.65_Rb_0.35_Fe_2_As_2_ investigated here, and for the closely related system Ba_1−*x*_K_*x*_Fe_2_As_2_, previous μSR measurements of *λ*(*T*) revealed a nodeless multi-gap SC state^2^,^3^, in agreement with angle-resolved photoemission spectroscopy (ARPES) measurements^1^,^30^,^31^. In this article, we report on μSR studies of *λ*(0) and of the temperature dependence of Δ*λ*^−2^ in optimally doped Ba_0.65_Rb_0.35_Fe_2_As_2_ under hydrostatic pressures. This system exhibits the highest *T*_c_≃37 K among the extensively studied 122 family of Fe-HTSs. We observe that while *T*_c_ stays nearly constant on application of pressure, *λ*(0) decreases substantially. In view of previous works in another 122 compound that reported a sharp peak of *λ*(0) at a quantum critical point^32^, we interpret the observed suppression of *λ*(0) as evidence that pressure moves the system away from a putative quantum critical point in Ba_0.65_Rb_0.35_Fe_2_As_2_. More importantly, we find a qualitative change in the low-temperature behaviour of Δ*λ*^−2^(*T*) as pressure is increased. While at *p*=0 an exponential suppression characteristic of a nodeless superconductivity is observed, for *p*=2.25 GPa a clear power-law behaviour is found. Because pressure does not affect the impurity concentration, which could promote power-law behaviour even for a nodeless system^33^, our findings are suggestive of a nodeless to nodal SC transition. Our fittings to microscopic models reveal that this behaviour is more compatible with a *d*-wave state rather than an *s*^+−^ state with accidental nodes, suggesting that pressure promotes a change in the pairing symmetry.

## Results

### Probing the vortex state as a function of pressure

**Figure 1:**
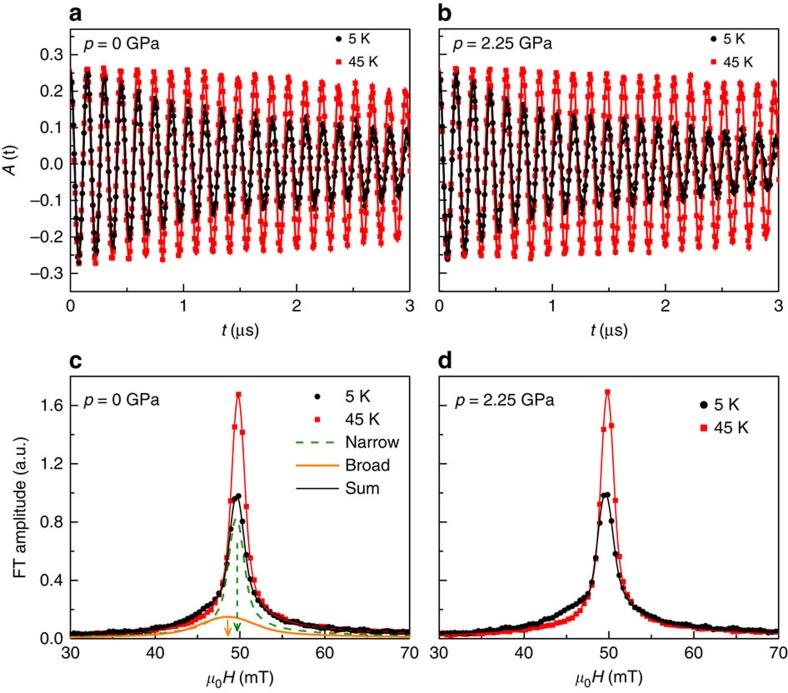
μSR time spectra and the corresponding Fourier transforms. The spectra for Ba_0.65_Rb_0.35_Fe_2_As_2_ are obtained above (45 K) and below (5 K) *T*_c_ (after field cooling the sample from above *T*_c_): (**a**,**c**) *p*=0 GPa and (**b**,**d**) *p*=2.25 GPa. Error bars are the s.e.m. in about 10^6^ events. The error of each bin count *n* is given by the s.d. of *n*. The errors of each bin in *A*(*t*) are then calculated by s.e. propagation. The solid lines in **a** and **b** represent fits to the data by means of equation (3). The solid lines in **c** and **d** are the Fourier transforms of the fitted time spectra. The dashed and solid arrows indicate the first moments for the signals of the pressure cell and the sample, respectively.

Figure 1a,b exhibit the transverse-field μSR time spectra for Ba_0.65_Rb_0.35_Fe_2_As_2_, measured at ambient *p*=0 GPa and maximum applied pressure *p*=2.25 GPa, respectively. The spectra above (45 K) and below (1.7 K) the SC transition temperature *T*_c_ are shown. Above *T*_c_ the oscillations show a small relaxation due to the random local fields from the nuclear magnetic moments. Below *T*_c_ the relaxation rate strongly increases with decreasing temperature due to the presence of a non-uniform local magnetic field distribution as a result of the formation of a flux-line lattice in the SC state. Figure 1c,d show the Fourier transforms of the μSR time spectra shown in Fig. 1a,b, respectively. At *T*=5 K the narrow signal around *μ*_0_*H*_ext_=50 mT (Fig. 1c,d) originates from the pressure cell, while the broad signal with a first moment *μ*_0_*H*_int_<*μ*_0_*H*_ext_, marked by the solid arrow in Fig. 1c, arises from the SC sample.

**Figure 2:**
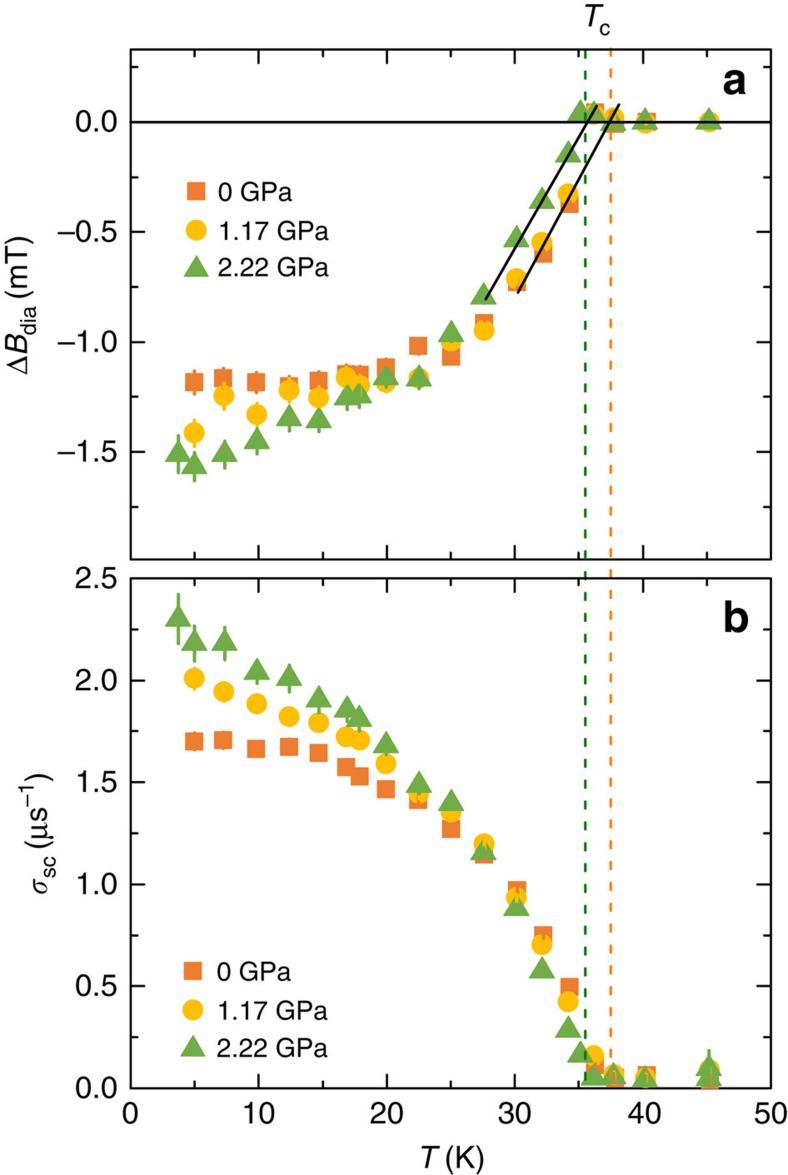
Diamagnetic shift and the relaxation rate. The temperature dependence of the diamagnetic shift Δ*B*_dia_=*μ*_0_[*H*_int,SC_−*H*_int,NS_] (**a**) and the muon spin relaxation rate *σ*_sc_ (**b**) for Ba_0.65_Rb_0.35_Fe_2_As_2_, measured in a magnetic field of *μ*_0_*H*=50 mT. The dashed vertical lines denote *T*_c_ for *p*=0 and 2.22 GPa. The error bars represent the s.d. of the fit parameters.

Below *T*_c_ a large diamagnetic shift of *μ*_0_*H*_int_ experienced by the muons is observed at all applied pressures. This is evident in Fig. 2a, where we plot the temperature dependence of the diamagnetic shift Δ*B*_dia_=*μ*_0_[*H*_int,SC_−*H*_int,NS_] for Ba_0.65_Rb_0.35_Fe_2_As_2_ at various pressures, where *μ*_0_*H*_int,SC_ denotes the internal field measured in the SC state and *μ*_0_*H*_int,NS_ the internal field measured in the normal state at 45 K. Note that *μ*_0_*H*_int,NS_ is temperature independent. This diamagnetic shift indicates the bulk character of superconductivity and excludes the possibility of field-induced magnetism^34^ in Ba_0.65_Rb_0.35_Fe_2_As_2_ at all applied pressures. The SC transition temperature *T*_c_ is determined from the intercept of the linearly extrapolated Δ*B*_dia_ curve to its zero line (we used the same criterium for determination of *T*_c_ from Δ*B*_dia_(*T*) as from the susceptibility data *χ*_m_(*T*), presented in Supplementary Fig. 1a). It is found to be *T*_c_=36.9(7) K and 35.9(5) K for *p*=0 and 2.25 GPa, respectively. The ambient pressure value of *T*_c_ is in perfect agreement with *T*_c_=36.8(5) K obtained from susceptibility and specific heat measurements (Supplementary Note 1 and Supplementary Figs 1a,b and 2). At the highest pressure of *p*=2.25 GPa applied, *T*_c_ decreases only by ≃1 K, indicating only a small pressure effect on *T*_c_ in Ba_0.65_Rb_0.35_Fe_2_As_2_. The temperature dependence of the muon spin depolarization rate *σ*_s*c*_ of Ba_0.65_Rb_0.35_Fe_2_As_2_ in the SC state at selected pressures is shown in Fig. 2b; note that *σ*_sc_ is proportional to the second moment of the field distribution, which was extracted using the equations described in the Method section. At all applied pressures, below *T*_c_ the relaxation rate *σ*_sc_ starts to increase from zero with decreasing temperature due to the formation of the flux-line lattice (we note that no pressure-induced magnetism is observed in Ba_0.65_Rb_0.35_Fe_2_As_2_, as shown in Supplementary Note 2 and Supplementary Figs 3a,b and 4). It is interesting that the low-temperature value *σ*_sc_(5 K) increases substantially under pressure (Fig. 2b): *σ*_sc_(5 K) increases about 30% from *p*=0 to 2.25 GPa. Interestingly, the form of the temperature dependence of *σ*_sc_, which reflects the topology of the SC gap, changes as a function of pressure. The most striking change is in the low-temperature behaviour of *σ*_sc_(*T*). At ambient pressure *σ*_sc_(*T*) shows a flat behaviour below *T*/*T*_c_≃0.4, whereas the high-pressure data exhibit a steeper (linear) temperature dependence of *σ*_sc_(*T*) below *T*/*T*_c_≃0.4. We show in the following how these behaviours indicate the appearance of nodes in the gap function.

### Pressure-dependent magnetic penetration depth

To investigate a possible change of the symmetry of the SC gap, we note that *λ*(*T*) is related to the relaxation rate *σ*_sc_(*T*) by the equation^35^:


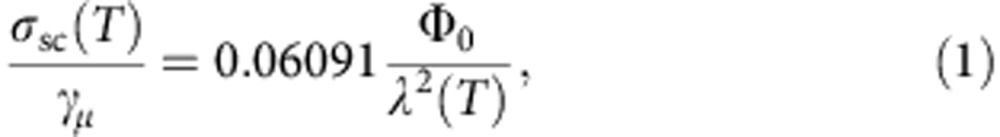


where *γ*_μ_ is the gyromagnetic ratio of the muon and Φ_0_ is the magnetic-flux quantum. Thus, the flat *T* dependence of *σ*_sc_ observed at *p*=0 for low temperatures (Fig. 2b) is consistent with a nodeless superconductor, in which *λ*^−2^(*T*) reaches its zero-temperature value exponentially. On the other hand, the linear *T* dependence of *σ*_sc_ observed at *p*=2.25 GPa (Fig. 2b) indicates that *λ*^−2^(*T*) reaches *λ*^−2^(0) linearly, which is characteristic of line nodes. This is the main result of this communication: pressure in an optimally doped Fe-HTS can tune a nodeless gap into a nodal gap. Although this qualitative analysis is robust, and independent of any fitting models for the gap function, it does not elucidate whether these nodes arise due to a nodal *s*^+−^ state or a *d*-wave state.

To proceed with a quantitative analysis, we consider the local (London) approximation (*λ*≫*ξ*, where *ξ* is the coherence length) and first employ the empirical α-model. The latter, widely used in previous investigations of the penetration depth of multiband superconductors^3^,^36^,^37^,^38^,^39^,^40^,^41^, assumes that the gaps occurring in different bands, besides a common *T*_c_, are independent of each other. Then, the superfluid density is calculated for each component separately^3^ and added together with a weighting factor. For our purposes a two-band model suffices yielding:


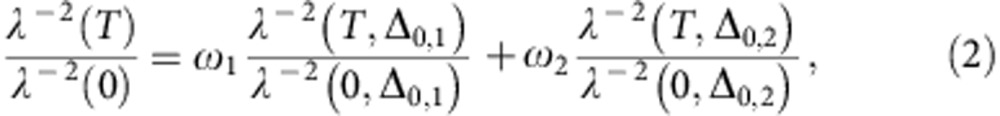


where *λ*(0) is the penetration depth at zero temperature, Δ_0,*i*_ is the value of the *i*-th SC gap (*i*=1, 2) at *T*=0 K and *ω*_i_ is the weighting factor, which measures their relative contributions to *λ*^−2^ (that is, *ω*_1_+*ω*_2_=1).

**Figure 3:**
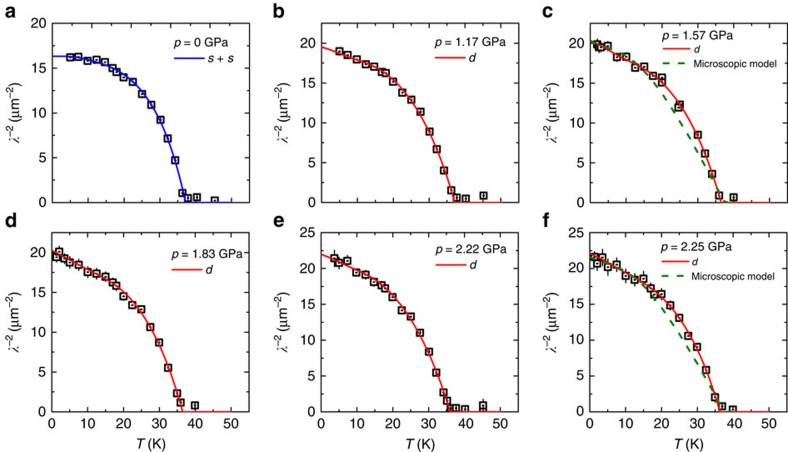
Inverse-squared magnetic penetration depth. The temperature dependence of *λ*^−2^ measured at various applied hydrostatic pressures for Ba_0.65_Rb_0.35_Fe_2_As_2_. The solid line for *p*=0 GPa corresponds to a two-gap *s*-wave model (**a**) and the solid lines for finite pressure represent a fits to the data using a multiband *d*-wave model (**b**–**f**). The dashed lines in **c** and **f** represent fits to the data using the microscopic model. The error bars are calculated as the s.e.m.

The results of this analysis are presented in Fig. 3a–f, where the temperature dependence of *λ*^−2^ for Ba_0.65_Rb_0.35_Fe_2_As_2_ is plotted at various pressures. We consider two different possibilities for the gap functions: either a constant gap, Δ_0,*i*_=Δ_*i*_, or an angle-dependent gap of the form Δ_0,*i*_=Δ_*i*_cos2*ϕ*, where *ϕ* is the polar angle around the Fermi surface. The resulting functions *λ*(*T*) are shown in the Methods section. The data at *p*=0 GPa are described remarkably well by two constant gaps, Δ_1_=2.7(5) meV and Δ_2_=8.4(3) meV. These values are in perfect agreement with our previous results^3^ and also with ARPES experiments^30^, pointing out that most Fe-HTSs exhibit two-gap behaviour, characterized by one large gap with 2Δ_2_/*k*_B_*T*_c_=7(2) and one small gap with 2Δ_1_/*k*_B_*T*_c_=2.5(1.5). In contrast to the case *p*=0 GPa, for all applied pressures *λ*^−2^(*T*) is better described by one constant gap and one angle-dependent gap, consistent with the presence of gap nodes, as inferred from our qualitative analysis. Note that a fitting to two angle-dependent gaps is inconsistent with the data.

**Figure 4:**
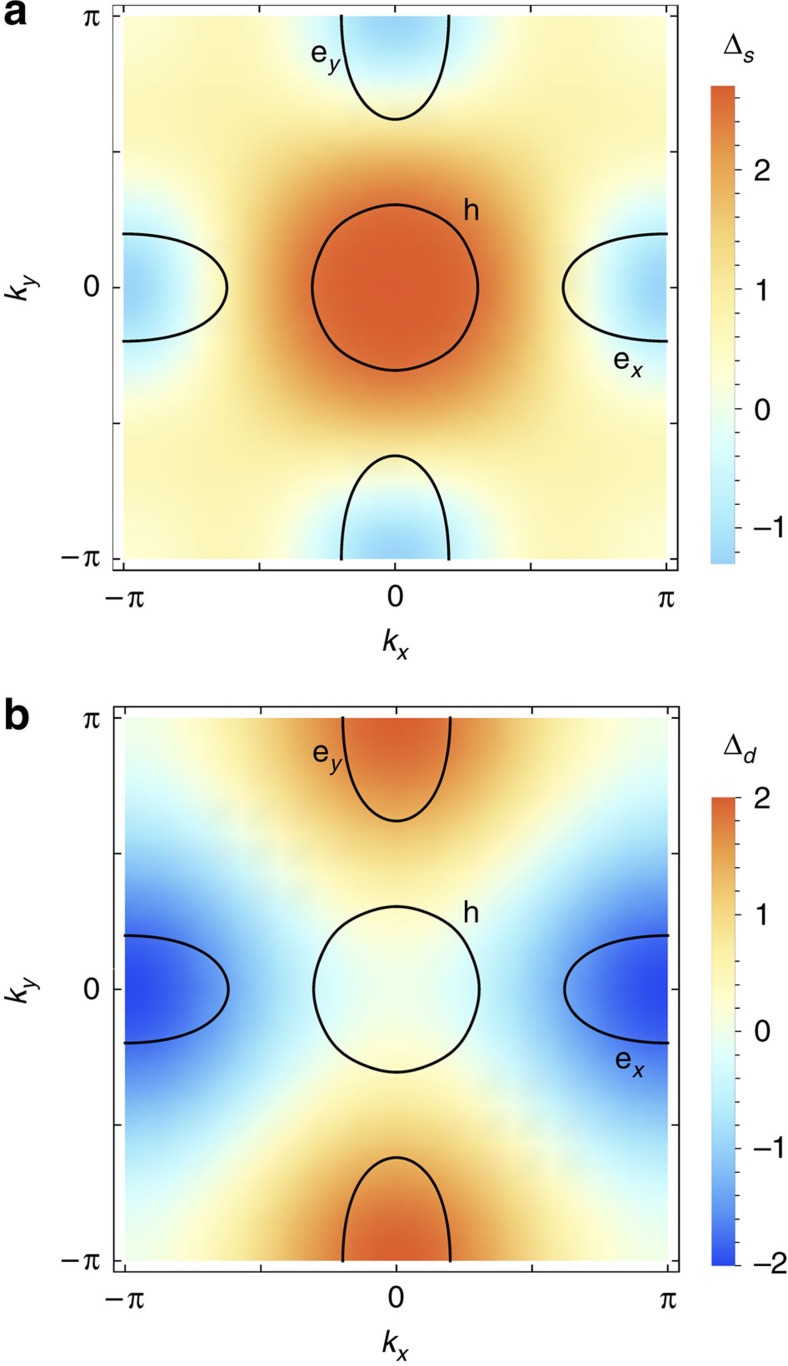
Schematic representation of the nodal *s*^+−^ and *d*-wave states. In both figures, a density plot of the gap function is superimposed to a representative Fermi surface consisting of a hole pocket (h) at the centre and an electron pocket (e) at the borders of the Brillouin zone. In the nodal *s*^+−^ states (**a**) the nodes are not enforced by symmetry (here they are located at the electron pockets). In the *d*-wave state (**b**) the nodes are enforced by symmetry to be on the diagonals of the Brillouin zone, and therefore can only cross the hole pockets.

To understand the implications of the fitting to a constant and an angle-dependent gap for finite pressures, we analyse the two different scenarios in which nodes can emerge: a nodal *s*^+−^ state (with gap functions of different signs in the hole and in the electron pockets) and a *d*-wave state. In the former, the position of the nodes are accidental, that is, not enforced by symmetry, while in the latter the nodes are enforced by symmetry to be on the Brillouin zone diagonals. Schematic representations of both scenarios are shown in Fig. 4, where a density plot of the gap functions is superimposed to the typical Fermi surface of the iron pnictides, consisting of one or more hole pockets at the centre of the Brillouin zone, and electron pockets at the border of the Brillouin zone. In this figure, we set the accidental nodes of the *s*^+−^ state to be on the electron pockets, as observed by ARPES in the related compound BaFe_2_(As_1−*x*_P_*x*_)_2_ (ref. 17). Note that in the *d*-wave state, while nodes appear in the hole pockets, the electron pockets have nearly uniform gaps. Thus, the fact that the fitting to the α-model gives a constant and an angle-dependent gap is consistent with a *d*-wave state.

To contrast the scenarios of a nodal *s*^+−^ gap and a *d*-wave gap, we consider a microscopic model (Supplementary Note 3) that goes beyond the simplifications of independent gap functions of the α-model discussed above. In this microscopic model, the fully coupled nonlinear gap equations are solved for a hole pocket h and two electron pockets e_1,2_, and the penetration depth is calculated at all temperatures (Supplementary Fig. 5). The free parameters are then the density of states of the pockets, the amplitude of the pairing interaction and the gap functions themselves (Supplementary Note 3). For simplicity, the anisotropies of the electron pockets are neglected, the Fermi velocities of the pockets are assumed to be nearly the same and the gaps are expanded in their leading harmonics. Thus, for the nodal *s*^+−^ state we have Δ_h_=Δ_h,0_ and 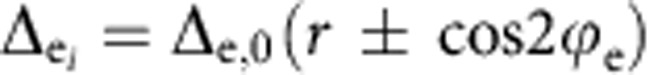, whereas for the *d*-wave state it follows that Δ_h_=Δ_h,0_ cos2*ϕ*_h_ and 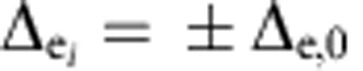. Note the difference in the position of the nodes in each case: while for the *d*-wave case they are always at *ϕ*_h_=±*π*/4, for the nodal *s*^+−^ the nodes exist only when *r*<1 at arbitrary positions 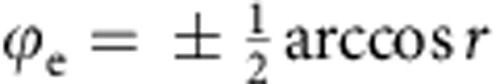. The results of the fittings for the pressures *p*=1.57 and 2.25 GPa imposing a nodal *s*^+−^ state are shown in Fig 3c,f (Supplementary Fig. 6a–c). Remarkably, we find in both cases that the best fit gives *r*→0. This extreme case is, within our model, indistinguishable from the fitting to the *d*-wave state, since in both cases the nodes are at *ϕ*=±*π*/4 (albeit in different Fermi pockets). We note that from the fits one cannot completely rule out the possibility of small but non-vanishing values of *r*. Therefore, at least within our model, a nodal *s*^+−^ state is compatible with the data only if the accidental nodes are fine tuned to lie either at or very close to the diagonals of the electron pockets for a broad pressure range. Since the position of the accidental nodes is expected to be sensitive to the topology of the Fermi surface, and consequently to pressure, it seems more plausible that the gap state is *d*-wave, since in that case the position of the gaps is enforced by symmetry to be along the diagonals of the hole pockets regardless of the value of the pressure (Supplementary Fig. 7a,b).

**Figure 5:**
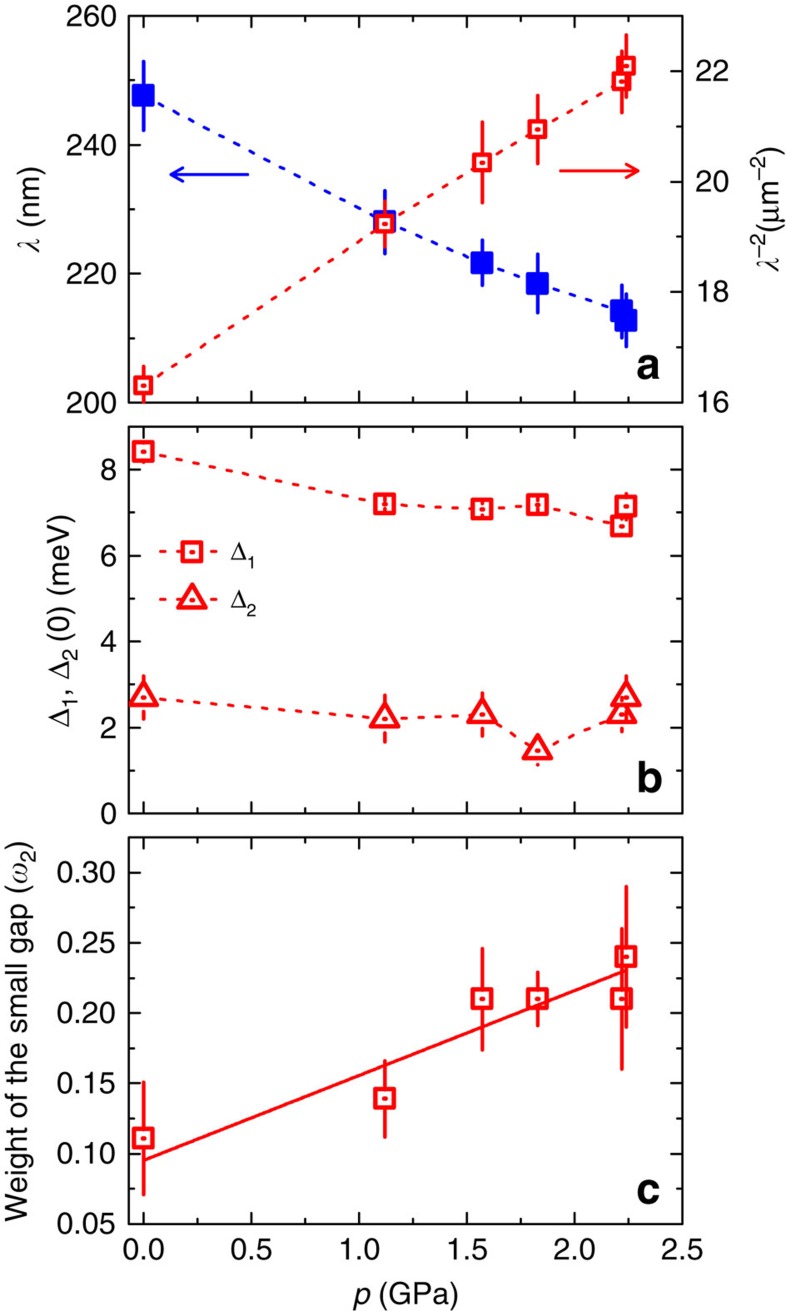
Pressure dependence of various quantities. The magnetic penetration depth *λ*(0) and *λ*^−2^(0) (**a**), the zero-temperature gap values Δ_1,2_(0) (**b**) as well as the relative weight *ω*_2_ of the small gap to the superfluid density (**c**) are plotted for Ba_0.65_Rb_0.35_Fe_2_As_2_ as a function of pressure. The error bars represent the s.d. of the fit parameters. The dashed lines are guides to the eyes and the solid lines represent linear fits to the data.

The pressure dependence of all the parameters extracted from the data analysis within the α-model are plotted in Fig. 5a–c. From Fig. 5a a substantial decrease of *λ*(0) with pressure is evident. At the maximum applied pressure of *p*=2.25 GPa the reduction of *λ*(0) is ∼15% compared with the value at *p*=0 GPa. Both Δ_1_ and Δ_2_ show a small reduction on increasing the pressure from *p*=0 to 1.17 GPa, while above *p*=1.17 GPa the gaps values stay constant. On the other hand, the relative contribution *ω*_2_ of the small gap to the superfluid density increases by approximately factor of 2 for the maximum applied pressure of *p*=2.25 GPa (Fig. 5c), indicating a spectral weight shift to the smaller gap. The parameters extracted from the microscopic model are discussed in Supplementary Note 3.

## Discussion

The main finding of our paper is the observation that pressure promotes a nodal SC gap in Ba_0.65_Rb_0.35_Fe_2_As_2_. This conclusion is model independent, as it relies on the qualitative change in the low-temperature behaviour of Δ*λ*^−2^ from exponential to linear in *T* on applied pressure. To our knowledge this is the first direct experimental demonstration of a plausible pressure-induced change in the SC gap structure in a Fe-HTS. Two possible gap structures could be realized at finite pressures: a nodal *s*^+−^ state and a *d*-wave state. In the first case, the change from nodeless *s*^+−^ to nodal *s*^+−^ is a crossover rather than a phase transition^42^,^43^, whereas in the latter it is an actual phase transition that could harbour exotic pairing states, such as *s*+*id* (refs 21, 23, 24) or *s*+*d* (ref. 44).

Additional results provide important clues of how pressure may induce either a nodal *s*^+−^ or a *d*-wave state. In the closely related optimally doped compound Ba_0.6_K_0.4_Fe_2_As_2_, Raman spectroscopy^27^, as well as theoretical calculations^21^,^20^, reveal a subdominant *d*-wave state close in energy to the dominant *s*^+−^ state. Pressure may affect this intricate balance, and tip the balance in favour of the *d*-wave state. On the other hand, theoretical calculations have shown that the pnictogen height is an important factor in determining the structure of the *s*^+−^ SC order parameter^18^,^45^. A systematic comparison of the quasiparticle excitations in the 1111, 122 and 111 families of Fe-HTSs showed that the nodal *s*^+−^ state is favoured when the pnictogen height decreases below a threshold value of ≃1.33 Å (ref. 46). Hydrostatic pressure may indeed shorten the pnictogen height and consequently modify the *s*^+−^ gap structure from nodeless to nodal. Although our fitting of the penetration depth data to both a microscopic model and an effective α-model suggest that the *d*-wave state is more likely to be realized than the nodal *s*^+−^ state, further quantitative calculations of the pressure effect are desirable to completely discard a nodal *s*^+−^ state.

Besides the appearance of nodes with pressure, another interesting observation is the reduction of *λ*(0) under pressure, despite the fact that *T*_c_ remains nearly unchanged. Interestingly, in the compound BaFe_2_As_2−*x*_P_*x*_, a sharp enhancement of *λ*(0) is observed as optimal doping is approached from the overdoped side^32^, which has been interpreted in terms of a putative quantum critical point (QCP) inside the SC dome^47^,^48^,^49^. In Ba_0.65_Rb_0.35_Fe_2_As_2_, if such a putative QCP is also present, pressure is likely to move the system away from the putative QCP, which, according to the results of BaFe_2_As_2−*x*_P_*x*_, would explain the observed suppression of the penetration depth at *T*=0. This scenario does not explain why *T*_c_ stays nearly constant under pressure, but this could be due to the intrinsic flatness of *T*_c_ around optimal doping in Ba_1−*x*_Rb_*x*_Fe_2_As_2_. Note that a similar behaviour for *λ*(0) and *T*_c_ with pressure has been recently observed in LaFeAsO_1−*x*_F_*x*_ (ref. 50), but interpreted in terms of the interplay between impurity scattering and pressure. To distinguish between these two scenarios, pressure-dependent studies of the quasiparticle mass in Ba_0.65_Rb_0.35_Fe_2_As_2_ are desirable to probe whether a putative QCP is present or not in this compound.

In conclusion, the zero-temperature magnetic penetration depth *λ*(0) and the temperature dependence of *λ*^−2^ were studied in optimally doped Ba_0.65_Rb_0.35_Fe_2_As_2_ by means of μSR experiments as a function of pressure up to 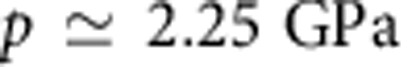. The SC transition temperature stays nearly constant under pressure, whereas a strong reduction of *λ*(0) is observed, possibly related to the presence of a putative quantum critical point. Our main result is the observation of a qualitative change in the low-temperature behaviour of Δ*λ*^−2^(*T*) from exponential to linear in the investigated Fe-based superconductor as pressure is increased. This most likely indicates that a nodal SC gap is promoted by hydrostatic pressure. Model calculations favour a *d*-wave over a nodal *s*^+−^-wave pairing as the origin for the nodal gap. The present results offer important benchmarks for the elucidation of the complex microscopic mechanism responsible for the observed non-universaltiy of the SC gap structure and of high-temperature superconductivity in the Fe-HTSs in general.

## Methods

### Sample preparation

Polycrystalline samples of Ba_0.65_Rb_0.35_Fe_2_As_2_ were prepared in evacuated quartz ampoules by a solid-state reaction method. Fe_2_As, BaAs and RbAs were obtained by reacting high-purity As (99.999 %), Fe (99.9%), Ba (99.9%) and Rb (99.95%) at 800, 650 and 500 °C, respectively. Using stoichiometric amounts of BaAs or RbAs and Fe_2_As, the terminal compounds BaFe_2_As_2_ and RbFe_2_As_2_ were synthesized at 950 and 650 °C, respectively. Finally, samples of Ba_1−*x*_Rb_*x*_Fe_2_As_2_ with *x*=0.35 were prepared from appropriate amounts of single-phase BaFe_2_As_2_ and RbFe_2_As_2_. The components were mixed, pressed into pellets, placed into alumina crucibles and annealed for 100 h under vacuum at 650 °C with one intermittent grinding. Powder X-ray diffraction analysis revealed that the synthesized samples are single-phase materials.

### Method for creation and measurement of high pressures

Pressures up to 2.4 GPa were generated in a double-wall piston-cylinder type of cell made of MP35N material, especially designed to perform μSR experiments under pressure^51^,^52^. As a pressure-transmitting medium Daphne oil was used. The pressure was measured by tracking the SC transition of a very small indium plate by a.c. susceptibility. The filling factor of the pressure cell was maximized. The fraction of the muons stopping in the sample was ∼40%.

### μSR experiment

Zero-field and transverse-field (TF) μSR experiments at ambient and under various applied pressures were performed at the μE1 beamline of the Paul Scherrer Institute, Switzerland, using the dedicated general purpose decay channel instrument (GPD) spectrometer, where an intense high-energy (*p*_μ_=100 MeVc^−1^) beam of muons is implanted in the sample through the pressure cell. A gas-flow ^4^He (base temperature ∼4 K) and a VARIOX cryostat (base temperature ∼1.3 K) were used. High-energy muons (*p*_μ_=100 MeVc^−1^) were implanted in the sample. Forward and backward positron detectors with respect to the initial muon spin polarization were used for the measurements of the μSR asymmetry time spectrum *A*(*t*). The typical statistics for both forward and backward detectors were 6 millions. All zero-field and transverse-field μSR experiments were performed by stabilizing the temperature in before recording the μSR time spectra. Note that a precise calibration of the GPD results was carried out at the πM3 beamline using the low-background GPS. The μSR time spectra were analysed using the free software package MUSRFIT^36^.

In a μSR experiment nearly 100% spin-polarized muons μ^+^ are implanted into the sample one at a time. The positively charged μ^+^ thermalize at interstitial lattice sites, where they act as magnetic microprobes. In a magnetic material the muon spin precesses in the local field *B*_μ_ at the muon site with the Larmor frequency *ν*_μ_=*γ*_μ_/(2*π*)*B*_μ_ (muon gyromagnetic ratio *γ*_μ_/(2*π*)=135.5 MHz T^−1^). By means of μSR important length scale of superconductor can be measured, namely the magnetic penetration depth *λ*. When a type II superconductor is cooled below *T*_c_ in an applied magnetic field ranging between the lower (*H*_c1_) and the upper (*H*_c2_) critical field, a vortex lattice is formed, which in general is incommensurate with the crystal lattice, and the vortex cores will be separated by much larger distances than those of the unit cell. Because the implanted muons stop at given crystallographic sites, they will randomly probe the field distribution of the vortex lattice. Such measurements need to be performed in a field applied perpendicular to the initial muon spin polarization (so called transverse-field configuration).

### Analysis of transverse-field-μSR data

Our zero-field μSR experiments (Supplementary Note 2) reveal a pressure-independent magnetic fraction of about 10% in the sample, caused by the presence of diluted Fe moments as discussed in previous μSR studies. The signal from the magnetically ordered parts vanishes within the first 0.2 μs. Thus, the fits of transverse-field data were restricted to times *t*>0.2 μs for all temperatures.

The transverse-field μSR data were analysed by using the following functional form^36^:


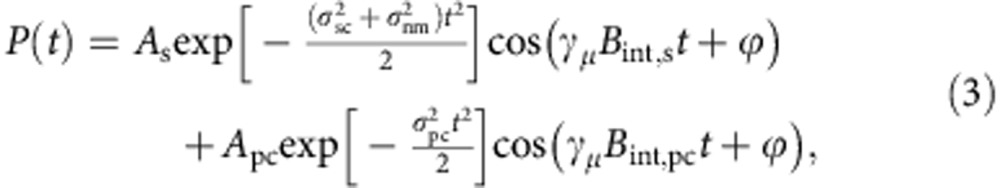


*A*_pc_ denote the initial assymmetries of the sample and the pressure cell, respectively. 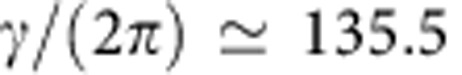 MHz T^−1^ is the muon gyromagnetic ratio, *ϕ* is the initial phase of the muon spin ensemble and *B*_int_ represents the internal magnetic field at the muon site. The relaxation rates *σ*_sc_ and *σ*_nm_ characterize the damping due to the formation of the vortex lattice in the SC state and of the nuclear magnetic dipolar contribution, respectively. In the analysis *σ*_nm_ was assumed to be constant over the entire temperature range and was fixed to the value obtained above *T*_c_, where only nuclear magnetic moments contribute to the muon relaxation rate *σ*. The Gaussian relaxation rate *σ*_p*c*_ reflects the depolarization due to the nuclear magnetism of the pressure cell. It can be seen from the Fourier transforms shown in Fig. 1c,d that the width of the pressure cell signal increases below *T*_c_. As shown previously^53^, this is due to the influence of the diamagnetic moment of the SC sample on the pressure cell, leading to a temperature-dependent *σ*_pc_ below *T*_c_. To consider this influence, we assume a linear coupling between *σ*_pc_ and the field shift of the internal magnetic field in the SC state: *σ*_pc_(*T*)=*σ*_pc_(*T*>*T*_c_)+*C*(*T*)(*μ*_0_*H*_int,NS_−*μ*_0_*H*_int,SC_), where *σ*_pc_(*T*>*T*_c_)=0.35 μs^−1^ is the temperature-independent Gaussian relaxation rate. *μ*_0_*H*_int,NS_ and *μ*_0_*H*_int,SC_ are the internal magnetic fields measured in the normal and in the SC state, respectively. As indicated by the solid lines in Fig. 1a–d, the μSR data are well described by equation (1). The solid lines in Fig. 1c,d are the Fourier transforms of the fitted curves shown in Fig. 1a,b. The model used describes the data rather well.

### Analysis of *λ*(*T*)

As pointed out in the manuscript, for polycrystalline samples the temperature dependence of the London magnetic penetration depth *λ*(*T*) is related to the muon spin depolarization rate *σ*_sc_(*T*) by equation (1) (see the main text). Equation (1) is valid when the separation between the vortices is smaller than *λ* and the applied field small with respect to the second critical field *B*_c2_. In this case according to the London model *σ*_sc_ is field independent^35^. Field-dependent measurements of *σ*_sc_ at ambient pressure was reported previously^3^. It was observed that first *σ*_sc_ strongly increases with increasing magnetic field until reaching a maximum at *μ*_0_*H*≃0.03 T and then above 0.03 T stays nearly constant up to the highest field (0.64 T) investigated. Such a behaviour is expected within the London model and is typical for polycrystalline HTSs^54^.

*λ*(*T*) was calculated within the local (London) approximation 
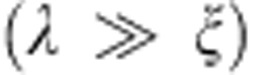
 by the following expression^36^,^37^:


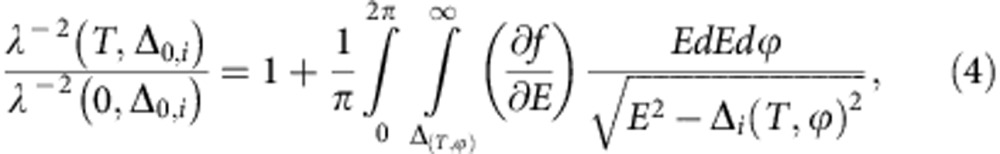


where *f*=[1+exp(*E*/*k*_B_*T*)]^−1^ is the Fermi function, *ϕ* is the angle along the Fermi surface and Δ_*i*_(*T*, *ϕ*)=Δ_0,*i*_Γ(*T*/*T*_c_)*g*(*ϕ*) (Δ_0,*i*_ is the maximum gap value at *T*=0). The temperature dependence of the gap is approximated by the expressions Γ(*T*/*T*_c_)=tanh{1.82[1.018(*T*_c_/*T*−1)]^0.51^} (ref. 38), while *g*(*ϕ*) describes the angular dependence of the gap and it is replaced by 1 for both an *s*-wave and an *s*+*s*-wave gap, and |cos(2*ϕ*)| for a *d*-wave gap^39^. The fitting of the *T* dependence of the penetration depth with α-model was performed using the library BMW^36^.

## Additional information

**How to cite this article:** Guguchia, Z. *et al*. Direct evidence for a pressure-induced nodal superconducting gap in the Ba_0.65_Rb_0.35_Fe_2_As_2_ superconductor. *Nat. Commun*. 6:8863 doi: 10.1038/ncomms9863 (2015).

## Supplementary Material

Supplementary Information Supplementary Figures 1-7, Supplementary Notes 1-3, and Supplementary References.
