# Supplementary material for: Direct evidence for a pressure-induced nodal superconducting gap in the Ba0.65Rb0.35Fe2As2 superconductor
Source: Nat Commun. 2015 Nov 9;6:8863. doi: 10.1038/ncomms9863 (PMC4667685; doi:10.1038/ncomms9863)
Supplement: Supplementary Information — Supplementary Figures 1-7, Supplementary Notes 1-3, and Supplementary References. [file ncomms9863-s1.pdf]

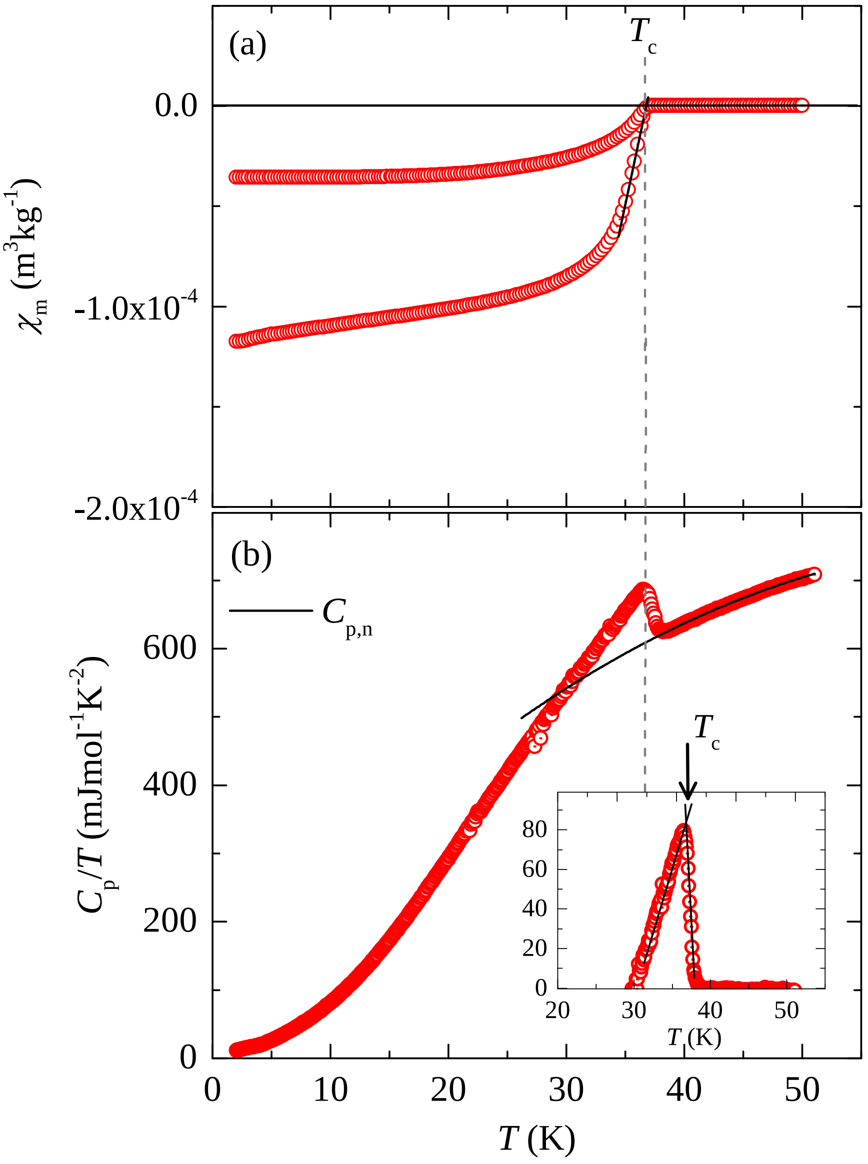

Supplementary Figure 1: **Magnetic susceptibility and specific heat**. (a) Temperature dependence of the zero-field cooled (ZFC)and field-cooled (FC) susceptibility χ_m obtained in an applied magnetic field of $\mu_0 H = 10$ mT for optimally doped $Ba_{0.65}Rb_{0.35}Fe_2As_2$. (b) Specific heat C_p/T as a function of temperature of $Ba_{0.65}Rb_{0.35}Fe_2As_2$. Inset shows the temperature dependence of the quantity $\Delta C_p/T$ with ΔC_p = (C_p - $C_{p,n}$), where $C_{p,n}$ represent the phonon dominated background. The arrow denotes the superconducting transition temperature T_c. Error bars indicate the statistical uncertainty of the data.

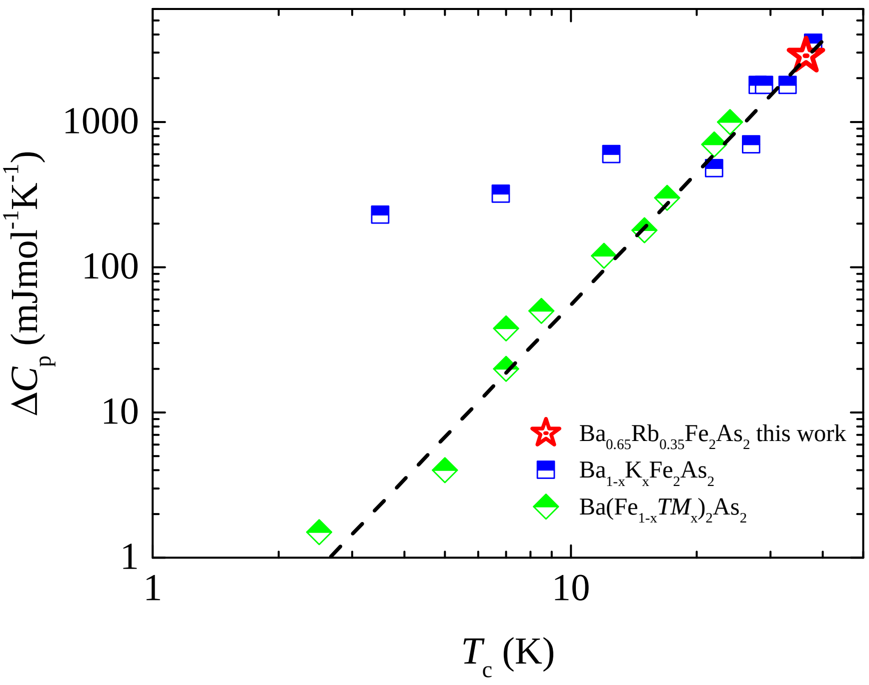

Supplementary Figure 2: **Specific heat scaling.** (a) Specific heat jump ΔC_p at the superconducting transition vs T_c for $Ba_{0.65}Rb_{0.35}Fe_2As_2$, plotted together with literature data for various FeAs-based superconductors. The line corresponds to $\Delta C_\mathrm{p} \propto T^3$ (after [1]).

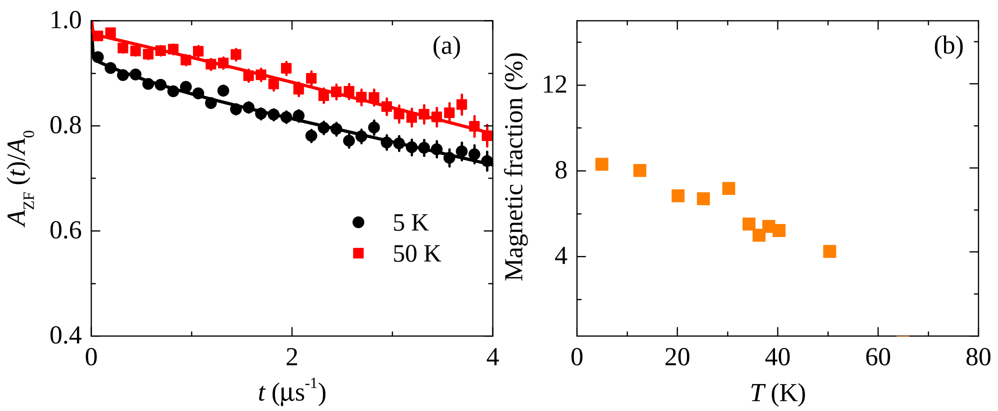

Supplementary Figure 3: **Magnetic signal at ambient pressure.** (a) The ZF-μSR time spectra for $Ba_{0.65}Rb_{0.35}Fe_2As_2$ recorded above and below T_c. Error bars indicate the statistical uncertainty of the data. The solid line represent the fits to the data by means of Supplementary Equation 1. (b) Temperature dependence of the magnetic fraction of $Ba_{0.65}Rb_{0.35}Fe_2As_2$, extracted from the ZF-$\mu$SR experiments. Error bars give the fit errors.

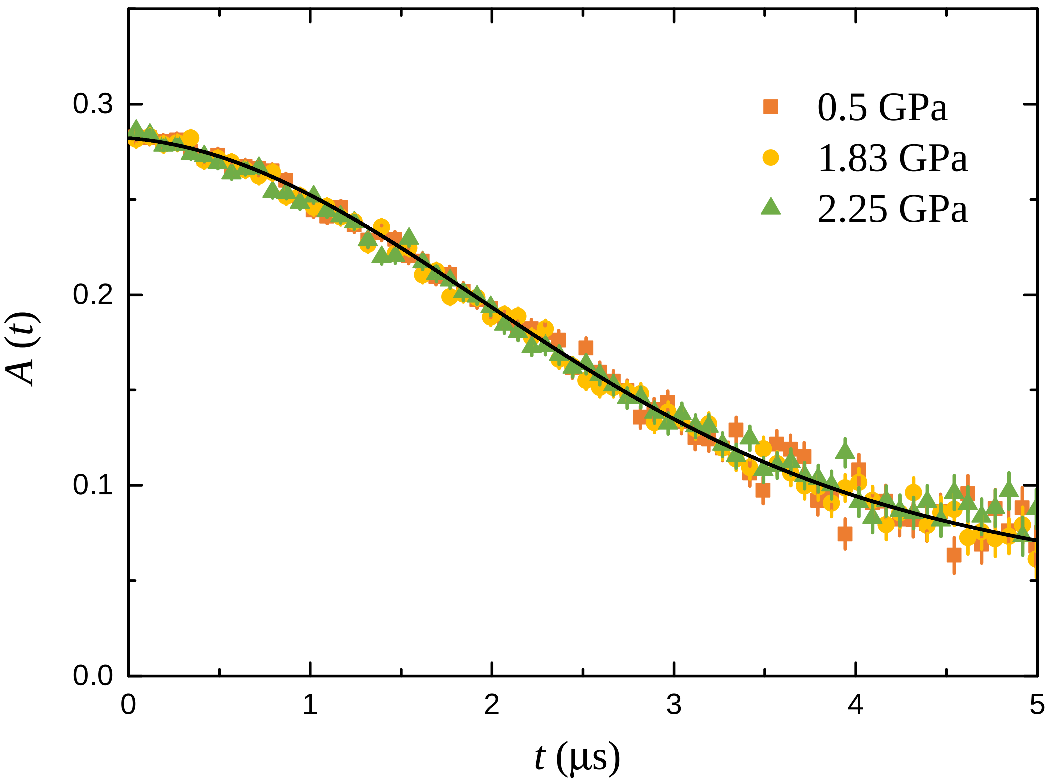

Supplementary Figure 4: **Pressure independent magnetic signal.** ZF-μSR time spectra for $Ba_{0.65}Rb_{0.35}Fe_2As_2$ at various applied pressures recorded at the base temperature $T = 1.4$ K. Error bars indicate the statistical errors of the data. The solid line represents the fit to the data by means of the sum of the Eq. (1) and a damped Kubo-Toyabe depolarization function to account for the pressure cell signal.

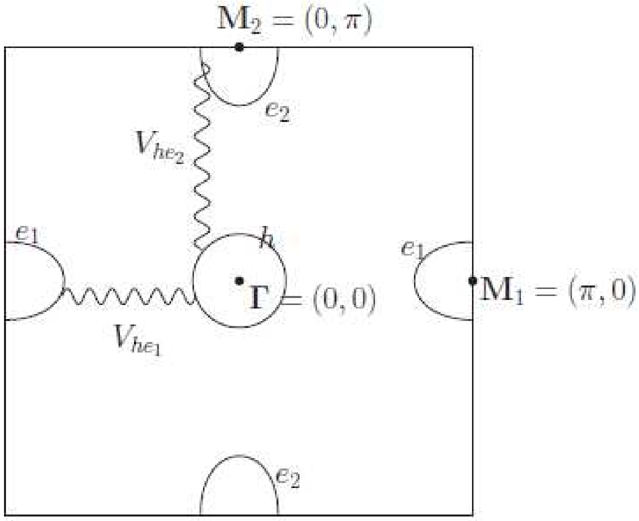

Supplementary Figure 5: **Three pocket model used in our calculations.** It is assumed that the system has one hole pocket h centered around $\Gamma = (0, 0)$ and two electron pockets e_1 and e_2 centered around $M_1 = (\pi, 0)$ and $M_2 = (0, \pi)$.

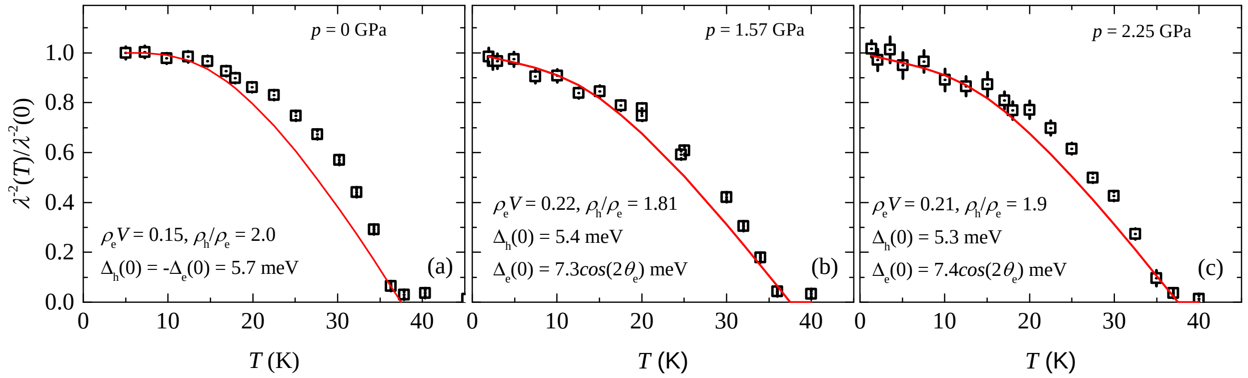

Supplementary Figure 6: **Analysis of the temperature dependence of the penetration depth using microscopic model.** The temperature dependence of $\lambda^{-2}(T)/\lambda^{-2}(0)$ measured at various applied hydrostatic pressures of $Ba_{0.65}Rb_{0.35}Fe_2As_2$. The square symbols are experimental data and the red curves are the theoretical functions. Error bars give the fit errors. (a) Fitting for the $P = 0$ data, which suggests a nodeless state. (b) and (c) Fitting for $P = 1.57$ GPa and $P = 2.25$ GPa. The fitting suggests that nodes exists on the two electron pockets at the angles $\theta_\mathrm{e} = \pm\pi/4$ and $\pm 3\pi/4$.

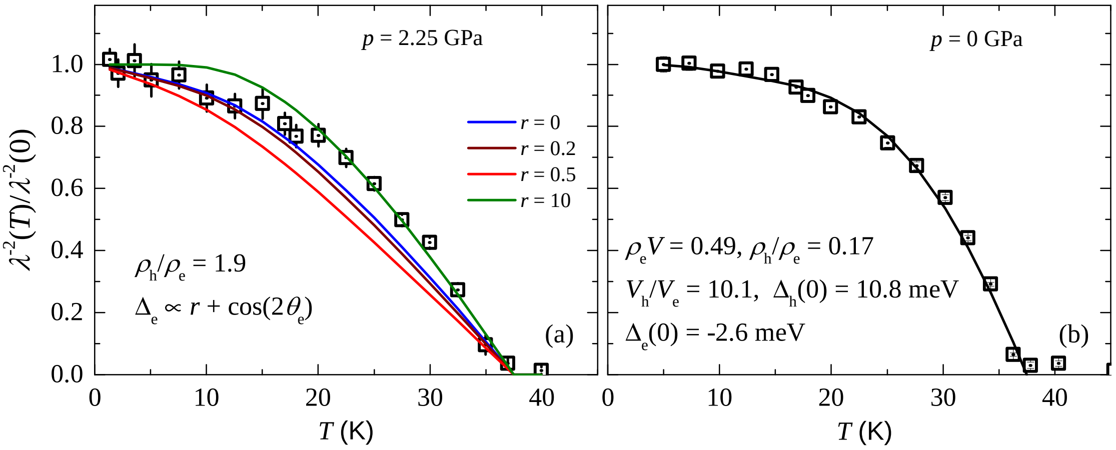

Supplementary Figure 7: **Effect of the electron pocket gap anisotropy on the penetration depth at $p = 2.25$ GPa.** (a) The electron gap is nodal if $r < 1$, and becomes nodeless if $r > 1$. The low temperature data clearly shows that the gap is nodal, but the data near T_c seems to be better described by a nodeless state. (b) Fitting for the zero pressure case with the Fermi velocity ratio $v_\mathrm{h}/v_\mathrm{e}$ being a free parameter. The fitting improves with respect to Fig. 6a, but the values of $v_\mathrm{h}/v_\mathrm{e}$ and $\rho_\mathrm{h}/\rho_\mathrm{e}$ seem to be too large or too small.

I. SUPPLEMENTARY NOTE 1: SAMPLE CHARACTERIZATION

The temperature dependence of the zero field-cooled (ZFC) and field-cooled (FC) diamagnetic susceptibility of $Ba_{0.65}Rb_{0.35}Fe_2As_2$ measured in a magnetic field of $\mu_0 H = 1$ mT is shown in Supplementary Figure 1a. From the diamagnetic response the SC transition temperature T_c is determined from the intercept of the linearly extrapolated zero-field cooled (ZFC) susceptibility curve with $\chi_m = 0$ line, and it is found to be $T_c = 36.8(5)$ K. The temperature-dependent heat capacity data for this sample plotted as C_p/T vs T is shown in Supplementary Figure 1b. The jump associated with the SC transitions is clearly seen. Here the anomaly at the transition has been isolated from the phonon dominated background by subtracting a second order polynomial $C_{p,n}$ fitted above T_c and extrapolated to lower temperature. The quantity $\Delta C_p/T$ with $\Delta C_p = (C_p$ - $C_{p,n})$ is presented as a function of temperature in the inset of Supplementary Figure 1b. Although there may be some uncertainty in using this procedure over an extended temperature range, the lack of appreciable thermal SC fluctuations, as evidenced by the mean-field-like form of the anomaly, means that there is very little uncertainty in the size of ΔC_p. Bud'ko et. al. [1] found that in many 122 Fe-based superconductors the specific heat jump ΔC_p at T_c follows the empirical trend, the so-called BNC scalling $\Delta C_p \propto T^3$. This has been interpreted as either originating from quantum critically or from strong impurity pair breaking. A violation of the BNC scaling was observed for $Ba_{1-x}K_xFe_2As_2$ for $x > 0.7$ [1] and in addition a change of the SC gap symmetry was observed. The specific heat jump data for $Ba_{0.65}Rb_{0.35}Fe_2As_2$ obtained in this work is added in Supplementary Figure 2 to the BNC plot taken from Ref. [1]. Our data point lies perfectly on the BNC line.

II. SUPPLEMENTARY NOTE 2: RESULTS OF THE ZERO-FIELD μSR EXPERIMENTS

It is well known that undoped $BaFe_2As_2$ is not superconducting at ambient pressure and undergoes a spin-density wave (SDW) transition of the Fe-moments far above T_c [2]. The SC state can be achieved either under pressure [3, 4] or by appropriate charge carrier doping of the parent compound [5], leading to a suppression of the SDW state. Magnetism, if present in the samples, must be taken into account in the TF-μSR data analysis. Therefore,

we have carried out ZF-μSR experiments above and below T_c to search for magnetism in $Ba_{0.65}Rb_{0.35}Fe_2As_2$. As an example, ZF-$\mu$SR spectra recorded at T = 5 K and 50 K of $Ba_{0.65}Rb_{0.35}Fe_2As_2$ are shown in Supplementary Figure 3a. There is no preccesion signal, indicating that there is no long-range magnetic order. On the other hand, we observed a significant drop of the asymmetry, taking place within 0.2 μs. This is caused by the presence of diluted Fe moments as discussed in previous μSR studies [6]. In order to quantify the magnetic fraction, the ZF-μSR data were analyzed by the following function:

$$A_{\rm ZF}(t) = \Omega A_0 \left[\frac{2}{3}e^{-\lambda_T t} + \frac{1}{3}e^{-\lambda_L t}\right] \\ +(1-\Omega)A_0\left[\frac{1}{3}+\frac{2}{3}(1-\sigma^2t^2-\Lambda t)e^{(-\frac{\sigma^2t^2}{2}-\Lambda t)}\right]. \quad (1)$$

the first and the second terms describe the magnetic and nonmagnetic part of the signals, respectively. A_0 is the initial asymmetry, Ω is the magnetic volume fraction, and $\lambda_{\rm T}$ ($\lambda_{\rm L}$) is the transverse (longitudinal) depolarization rate of the μSR signal, arising from the magnetic part of the sample. The second term describing the paramagnetic part of the sample is the combination of a Lorentzian and a Gaussian Kubo-Toyabe depolarization functions [7, 8]. σ and Λ are the depolarization rates due to the nuclear dipole moments and randomly oriented diluted local electronic moments, respectively. The temperature dependence of the magnetic fraction obtained for $Ba_{0.65}Rb_{0.35}Fe_2As_2$ is plotted in Supplementary Figure 3b. The magnetic fraction at the base temperature was found to be only 8 %. Bearing in mind that the signal from the magnetically ordered parts vanishes within the first 0.2 μs in the whole temperature region, the analysis of transverse field data was restricted to times $t >$ 0.2 μs.

Supplementary Figure 4 shows the ZF-μSR time spectra for $Ba_{0.65}Rb_{0.35}Fe_2As_2$ at various applied pressures. The ZF relaxation rate stays nearly unchanged between p = 0 GPa and 2.25 GPa, implying that there is no sign of pressure induced magnetism in this system.

III. SUPPLEMENTARY NOTE 3: MICROSCOPIC MODEL FOR ANALYZING THE PENETRATION DEPTH DATA OF $BA_{0.65}RB_{0.35}FE_2AS_2$

Model for s^{+-} pairing:

As a minimal model that accounts for the different superconducting states of the iron

pnictides (nodeless s^{+-}, nodal s^{+-}, and d-wave), we consider a two-dimensional system with three isotropic Fermi pockets [9]: one hole pocket h centered around $\Gamma = (0,0)$ and two electron pockets e_1 and e_2 centered around $M_1 = (\pi, 0)$ and $M_2 = (0, \pi)$ (see Supplementary Figure 5). To describe the s^{+-} state, the pairing interaction between the hole pocket h and the electron pocket e_1 is assumed to be angular dependent with the form:

$$V_{\mathrm{he_1}} = V_0(r - \cos 2\phi) h^\dagger_\uparrow(\mathbf{k}) h^\dagger_\downarrow(-\mathbf{k}) e_{1\downarrow}(-\mathbf{p}) e_{1\uparrow}(\mathbf{p}) + h.c. \ , \tag{2}$$

where ϕ is the polar angle measured relative to the center of the electron pocket, V_0 is the interaction energy scale, and r is the relative amplitude of the angular-independent and the angular-dependent pairing interactions. Due to the tetragonal symmetry of the system, the pairing interaction between h and e_2 is:

$$V_{\mathrm{he_2}} = V_0(r + \cos 2\phi) h^\dagger_\uparrow(\mathbf{k}) h^\dagger_\downarrow(-\mathbf{k}) e_{2\downarrow}(-\mathbf{p}) e_{2\uparrow}(\mathbf{p}) + h.c. \ . \tag{3}$$

Furthermore, to minimize the number of free parameters, we assume that the three pockets have the same Fermi velocity v_{f}, while the density of states can in principle be different $\rho_{\mathrm{h}}/\rho_{\mathrm{e}} = \eta$. Within this model, we obtain an s^{+-} state, where the SC gap of the hole pocket is a constant, Δ_{h}, and the gap on the electron pockets is of the form $\Delta_{\mathrm{e_1}} = \Delta_{\mathrm{e}}(r - \cos 2\phi)$ and $\Delta_{\mathrm{e_2}} = \Delta_{\mathrm{e}}(r + \cos 2\phi)$. Accidental nodes appear in the electron pockets if $r < 1$. Introducing the energy cutoff Λ_{c}, we can write down the corresponding BCS-like gap equations:

$$\Delta_{\mathrm{h}} = -\rho_{\mathrm{e}} V_0 \Delta_{\mathrm{e}} \int_{-\Lambda_{\mathrm{c}}}^{\Lambda_{\mathrm{c}}} \mathrm{d}\epsilon \int \frac{\mathrm{d}\phi}{2\pi} \left(\frac{(r+\cos 2\phi)^2}{2E_{\mathrm{e_1}}(\mathbf{k})} \tanh \frac{\beta E_{\mathrm{e_1}}(\mathbf{k})}{2} + \frac{(r-\cos 2\phi)^2}{2E_{\mathrm{e_2}}(\mathbf{k})} \tanh \frac{\beta E_{\mathrm{e_2}}(\mathbf{k})}{2} \right) \tag{4}$$

$$\Delta_{\mathrm{e}} = -\rho_{\mathrm{h}} V_0 \Delta_{\mathrm{h}} \int_{-\Lambda_{\mathrm{c}}}^{\Lambda_{\mathrm{c}}} \frac{\mathrm{d}\epsilon}{2E_{\mathrm{h}}(\mathbf{k})} \tanh \frac{\beta E_{\mathrm{h}}(\mathbf{k})}{2} \tag{5}$$

where $E_{\mathrm{e_1}}(\mathbf{k})$, $E_{\mathrm{e_2}}(\mathbf{k})$, and $E_{\mathrm{h}}(\mathbf{k})$ are the quasi-particle energy dispersions:

$$E_{\mathrm{e_1}}(\mathbf{k}) = \sqrt{\epsilon_{\mathrm{e}}^2 + \Delta_{\mathrm{e}}^2 (r - \cos 2\phi)^2} \ , \quad E_2(\mathbf{k}) = \sqrt{\epsilon_{\mathrm{e}}^2 + \Delta_{\mathrm{e}}^2 (r + \cos 2\phi)^2} \ , \quad E_{\mathrm{h}}(\mathbf{k}) = \sqrt{\epsilon_{\mathrm{h}}^2 + \Delta_{\mathrm{h}}^2} \ .$$

To determine T_{c}, we linearize the gap equations, yielding:

$$\begin{cases} \Delta_{\mathrm{h}} = -\Delta_{\mathrm{e}} \rho_{\mathrm{e}} V_0 (2r^2+1) \displaystyle\int_0^{\Lambda_{\mathrm{c}}} \frac{\mathrm{d}\epsilon}{\epsilon} \tanh \frac{\beta_{\mathrm{c}}\epsilon}{2} \\ \Delta_{\mathrm{e}} = -\Delta_{\mathrm{h}} \eta \rho_{\mathrm{e}} V_0 \displaystyle\int_0^{\Lambda_{\mathrm{c}}} \frac{\mathrm{d}\epsilon}{\epsilon} \tanh \frac{\beta_{\mathrm{c}}\epsilon}{2} \end{cases} \Longrightarrow \rho_{\mathrm{e}} V_0 = \left[\sqrt{\eta(2r^2+1)} \int_0^{\Lambda_{\mathrm{c}}} \frac{\mathrm{d}\epsilon}{\epsilon} \tanh \frac{\beta_{\mathrm{c}}\epsilon}{2} \right]^{-1}$$

To perform the fitting, we set T_c to be fixed, and set the energy cutoff $\Lambda_\mathrm{c} = 86\mathrm{meV}$ (the results do not depend significantly on the choice of the cutoff). This provides a constraint on $\rho_\mathrm{e} V_0$, η, and r. When $T < T_\mathrm{c}$, the gaps are calculated based on the BCS Eqs. (4) and (5).

The expression for the penetration depth of a single-band system is:

$$\lambda_{\mu\mu}^{-2}(T) = \frac{4\pi}{cV}\sum_{\mathbf{k}}\left[\langle\frac{\partial^2\epsilon}{\partial k_\mu^2}\rangle + \left(\frac{\partial\epsilon}{\partial k_\mu}\right)^2\frac{\partial f}{\partial E_\mathrm{k}}\right] \to \frac{1}{V}\sum_{\mathbf{k}}\left(\frac{\partial\epsilon}{\partial k_\mu}\right)^2\left[\frac{\partial f}{\partial E_k} - \frac{\partial f}{\partial\epsilon_k}\right] ,$$

where f is the Fermi distribution function, ϵ is the energy of the non-interacting system, and E_k is the quasi-particle energy dispersion. Applying this formula to our three pocket model, we obtain

$$\begin{aligned}\lambda^{-2}(T) \propto& \rho_\mathrm{h}\frac{v_\mathrm{f}^2}{2}\int_{-\Lambda_\mathrm{c}}^{\Lambda_\mathrm{c}}\mathrm{d}\epsilon\left(\frac{\partial f}{\partial E_\mathrm{h}} - \frac{\partial f}{\partial\epsilon_\mathrm{h}}\right) + \rho_\mathrm{e}v_\mathrm{f}^2\int_{-\Lambda_\mathrm{c}}^{\Lambda_\mathrm{c}}\mathrm{d}\epsilon\int\frac{\mathrm{d}\phi}{2\pi}\cos^2\phi\left(\frac{\partial f}{\partial E_{\mathrm{e}_1}} - \frac{\partial f}{\partial\epsilon_\mathrm{e}}\right)\\ &+ \rho_\mathrm{e}v_\mathrm{f}^2\int_{-\Lambda_\mathrm{c}}^{\Lambda_\mathrm{c}}\mathrm{d}\epsilon\int\frac{\mathrm{d}\phi}{2\pi}\cos^2\phi\left(\frac{\partial f}{\partial E_{\mathrm{e}_2}} - \frac{\partial f}{\partial\epsilon_\mathrm{e}}\right)\\ \lambda^{-2}(T) \propto& \rho_\mathrm{e}v_\mathrm{f}^2\left[\frac{2+\eta}{2}\Big(1 - 2f(\Lambda_\mathrm{c})\Big) + \eta\int_0^{\Lambda_\mathrm{c}}\mathrm{d}\epsilon\frac{\partial f}{\partial E_\mathrm{h}} + 2\int_0^{\Lambda_\mathrm{c}}\mathrm{d}\epsilon\int\frac{\mathrm{d}\phi}{2\pi}\frac{\partial f}{\partial E_\mathrm{e}}\right] \end{aligned} \tag{6}$$

In the fittings, we will focus on the normalized penetration depth $\lambda^{-2}(T)/\lambda^{-2}(0)$.

Model for d-wave pairing:

To describe the d-wave superconducting state within our three band model, we consider the following form of the pairing interaction:

$$\begin{aligned} V_{\mathrm{he}_1} &= V_0(r - \cos 2\theta)h_\uparrow^\dagger(\mathbf{k})h_\downarrow^\dagger(-\mathbf{k})e_{1\downarrow}(-\mathbf{p})e_{1\uparrow}(\mathbf{p}) + h.c.\\ V_{\mathrm{he}_2} &= V_0(r + \cos 2\theta)h_\uparrow^\dagger(\mathbf{k})h_\downarrow^\dagger(-\mathbf{k})e_{2\downarrow}(-\mathbf{p})e_{2\uparrow}(\mathbf{p}) + h.c. \ . \end{aligned}$$

where θ is the angle around the hole pocket. The gap functions can then be written as:

$$\Delta_{\mathrm{e}_1} = -\Delta_{\mathrm{e}_2} = \Delta_\mathrm{e} \ , \quad \Delta_\mathrm{h}(\mathbf{k}) = \Delta_\mathrm{h}\cos 2\theta \ .$$

resulting in the BCS-like gap equations:

$$\begin{aligned} \Delta_\mathrm{h} &= 2\Delta_\mathrm{e}\rho_\mathrm{e}V_0\int_{-\Lambda_\mathrm{c}}^{\Lambda_\mathrm{c}}\frac{\mathrm{d}\epsilon}{2E_\mathrm{e}}\tanh\frac{\beta E_\mathrm{e}}{2}\\ \Delta_\mathrm{e} &= \Delta_\mathrm{h}\eta\rho_\mathrm{e}V_0\int_{-\Lambda_\mathrm{c}}^{\Lambda_\mathrm{c}}\mathrm{d}\epsilon\int\frac{\mathrm{d}\theta}{2\pi}\frac{\cos^2 2\theta}{2E_\mathrm{h}}\tanh\frac{\beta E_\mathrm{h}}{2} \end{aligned}$$

Here, $\eta = \rho_{\rm h}/\rho_{\rm e}$, $E_{\rm e} = \sqrt{\epsilon_{\rm e}^2 + \Delta_{\rm e}^2}$, and $E_{\rm h} = \sqrt{\epsilon^2 + \Delta_{\rm h}^2 \cos^2 2\theta}$. Repeating the same steps as for the s^{+-} case, we obtain the penetration depth:

$$\lambda^{-2}(T) \propto \rho_{\rm e} v_{\rm f}^2 \left[\frac{2+\eta}{2}(1 - 2f(\Lambda_{\rm c})) + \eta \int_0^{\Lambda_{\rm c}} {\rm d}\epsilon \int \frac{{\rm d}\theta}{2\pi} \frac{\partial f}{\partial E_{\rm h}} + 2 \int_0^{\Lambda_{\rm c}} {\rm d}\epsilon \frac{\partial f}{\partial E_{\rm e}} \right] \tag{7}$$

Comparing the expressions for the d-wave case to the expressions we derived for the s^{+-} case, Eqs. (4) and (6), we note that they can be mapped onto each other if $r = 0$. In this extreme case, changing $\eta_{\rm d} \to 4/\eta_{\rm s}$, $V_{0,{\rm d}} \to \eta V_{0,{\rm s}}/2$, and $\Delta_{\rm h} \leftrightarrow \Delta_{\rm e}$ leads to the same gap equations and penetration depth expression. With these replacements, both s and d pairing give the same $\lambda^{-2}(T)/\lambda^{-2}(0)$. Therefore, we conclude that the penetration depth cannot distinguish between nodal-s^{+-} and d-wave if the nodal-s^{+-} is the extreme case with $r = 0$.

Fitting Results:

We now fit the experimental data $\lambda^{-2}(T)/\lambda^{-2}(0)$ of optimally-doped $Ba_{1-x}Rb_xFe_2As_2$ to find the values of $\rho_{\rm e} V_0$, η, and r for different pressures. Note that the value of $T_{\rm c}$ imposes another constraint on these three parameters, as explained above. Supplementary Figures 6a, b and c show the fitting for the s^{+-} model for $P = 0$, $P = 1.57$ GPa, and $P = 2.25$ GPa, respectively. For the $P = 0$ case, we find equal gap amplitudes and no nodes, as seen by ARPES experiments in the related compound $Ba_{1-x}K_xFe_2As_2$. We see that the fitting is not as good in the region immediately below $T_{\rm c}$. We will discuss this issue in more details below. For the pressurized samples, the fitting is overall better and indicates a nodal state ($r < 1$). The value of the density of states ratio $\rho_{\rm h}/\rho_{\rm e}$ is little affected by pressure (as expected, since no charge carriers are introduced), and is consistent with the value of a nearly compensated metal.

Surprisingly, the best fittings for both the $P = 1.57$ GPa and $P = 2.25$ GPa cases give $r = 0$, where the nodes on the electron pockets are fixed at $\theta = \pm\pi/4$. This is a very special case of the accidentally nodal s^{+-} state, since by symmetry there is no reason for r to vanish. To make this point more transparent, in Supplementary Figure 7a we plot the non-zero pressure data and the theoretical urves for the penetration depth for various values of r – keeping all the other parameters constant. Clearly, $0 < r < 1$ gives worst fittings than $r = 0$. What we also found is that $r = 10$ – i.e. a nodeless superconducting state – describes the data better near $T_{\rm c}$, on the expense of a very bad fitting at low temperatures – where the nodal behavior is evident.

As we discussed in the previous section, a nodal-s^{+-} state with $r = 0$ is indistinguishable – for fitting purposes – from a d-wave state. Since there is no symmetry reason to have $r = 0$ in our simple model, or even $r \ll 1$ over a wide pressure range, we interpret this result as an indirect indication that a d-wave state is more likely to be the state of the pressurized samples.

Finally, we comment on the difficulty of the fittings to capture the behavior near T_c – particularly for the sample at ambient pressure (see Supplementary Figure 6a). One reason could be the presence of inhomogeneities, which would require a distribution of gaps to be taken into account, instead of a single gap value. Another reason could be related to our choice of fixing the Fermi velocities to be the same for both the electron and hole pockets. To investigate this possibility, we lift this restriction and allow $v_\mathrm{h}/v_\mathrm{e}$ to also be a fitting parameter. The result is shown in Supplementary Figure 7b. Clearly, we obtain a better fitting, but not only $\rho_\mathrm{e} V_0$ is relatively large, but the ratios $\rho_\mathrm{h}/\rho_\mathrm{e}$ and $v_\mathrm{h}/v_\mathrm{e}$ are very large or very small, which is difficult to reconcile with the Fermi surface of these materials. Most likely, additional pockets are necessary to capture the full temperature dependence of the penetration depth. Nevertheless, our microscopic model provides results that agree with those obtained from the α-model fitting, particularly in the low-temperature regime, suggesting that a d-wave state is more likely to be realized than a nodal s^{+-} state.

IV. SUPPLEMENTARY REFERENCES

[1] Bud'ko, S.L., Ni, Ni., and Canfield, Paul C. Jump in specific heat at the superconducting transition temperature in $Ba(Fe_{1-x}Co_x)_2As_2$ and $Ba(Fe_{1-x}Ni_x)_2As_2$. *Phys. Rev. B* **79**, 220516(R) (2009).

[2] Huang, Q. *et al.* Neutron-Diffraction Measurements of Magnetic Order and a Structural Transition in the Parent $BaFe_2As_2$ Compound of FeAs-Based High-Temperature Superconductors. *Phys. Rev. Lett.* **101**, 257003 (2008).

[3] Torikachvili, M.S., Bud'ko, S.L., Ni, N., and Canfield, P.C. Pressure Induced Superconductivity in $CaFe_2As_2$. *Phys. Rev. Lett.* **101**, 057006 (2008).

[4] Miclea, C.F. *et al.* Evidence for a reentrant superconducting state in $EuFe_2As_2$ under pressure *Phys. Rev. B* **79**, 212509 (2009).

[5] Zhao, J. *et al.* Structural and magnetic phase diagram of $CeFeAsO_{1-x}F_x$ and its relation to high-temperature superconductivity. *Nature Materials* **7**, 953-959 (2008).

[6] Khasanov, R. *et al.* Two-Gap Superconductivity in $Ba_{1-x}K_xFe_2As_2$: A Complementary Study of the Magnetic Penetration Depth by Muon-Spin Rotation and Angle-Resolved Photoemission. *Phys. Rev. Lett.* **102**, 187005 (2009).

[7] Kubo, R., and Toyabe, T. *Magnetic Resonance and Relaxation* (North Holland, Amsterdam, 1967).

[8] Hayano, R.S., Uemura, Y.J., Imazato, J., Nishida, N., Yamazaki, T., and Kubo, R. Zero-and low-field spin relaxation studied by positive muons. *Phys. Rev. B* **20**, 851-859 (1979).

[9] Kang, J., Kemper, A.F., and Fernandes, R. M. Manipulation of Gap Nodes by Uniaxial Strain in Iron-Based Superconductors. *Phys. Rev. Lett.* **113**, 217001 (2014).
